# Supplementary material for: Patterns of Intron Gain and Loss in Fungi
Source: PLoS Biol. 2004 Nov 30;2(12):e422. doi: 10.1371/journal.pbio.0020422 (PMC532390; doi:10.1371/journal.pbio.0020422)
Supplement: Table S1 — Also available at http://genes.mit.edu/NielsenEtAl/. (4.3 MB ZIP). [file pbio.0020422.st001.zip › NielsenEtAl/html/539.html]

AN8277.1.NCU01652.1.MG07195.1.FG01417.1


```
 CLUSTAL W (1.82) Multiple Sequence Alignments - Introns Inserted


Sequence 1: AN8277.1	437 aa
Sequence 2: FG01417.1	454 aa
Sequence 3: NCU01652.1	454 aa
Sequence 4: MG07195.1	456 aa
Alignment Length: 458 aa
Number Identitical Residues: 307 aa
Alignment Score (without introns) 13437


MG07195.1 	-MSDLSKNFETLQLHAG2QQPDPATNSRAVPIYATT0SFVFNDSAHGARLFGLKQFGNIY
NCU01652.1	----MSHHFETLQVHAG2QEPDPATKSRAVPIYATS0SYVFNDSAHGARLFGLKEFGNIY
FG01417.1 	MSEELSKNFETLQLHAG2AEIDPTTRSRAVPIYATT0SYAFKDSAHGARLFGLKEFGNIY
AN8277.1  	MSDPSPKRFETLQLHAG2QEPDPATNSRAVPIYATT0SYTFNDSAHGARLFGLKEFGNIY
          	  .  .:.*****:***  : **:*.*********: *:.*:************:*****

MG07195.1 	SRLAN~PTVDVFEKRVAALEGGVAAVATASGQAAQFMTILALAHAGDNIVSTSNLYGGTY
NCU01652.1	SRIMN2PTVDVFEKRIAALEGGLAAIATASGQAAQFMAISGVACAGDNIVSTSNLYGGTY
FG01417.1 	SRIMN0PTVDVFEKRIAALEGGVAALAASSGQAAQFLAISTLAQAGDNIVSTSNLYGGTY
AN8277.1  	SRIMN0PTVDVFEKRIAALEGGVAAVAASSGQAAQFMAISALAHAGDNIVSTSNLYGGTY
          	**: * *********:******:**:*::*******::*  :* ****************

MG07195.1 	NQFKVMFPRLGIQTKFVDGDKPEDLIAAIDDKTKAVYLESIGNPRYNIPDFEKITKLAHE
NCU01652.1	NQLKVMLPRYGIQTKFVTGDKPEDLAAAIDDKTKAVYLESIGNPRYNIPGFEAITKLAHE
FG01417.1 	NQFKVFFPRLGIKTKFVDGDKPEDIAAAIDDKTKAVYVESIGNPKYNIPDLEAISKAAHE
AN8277.1  	NQFKVLFPRLGITTKFVQGDKAEDIAAAIDDRTKAVYVETIGNPRYNVPDFEVIAKVAHE
          	**:**::** ** **** ***.**: *****:*****:*:****:**:*.:* *:* ***

MG07195.1 	KGVPVV0VDNTFGAGGYFVRPIEHGADIVVHSATKWIGGHGTTIGGIIVDSGKFDWGANS
NCU01652.1	KGVPVI0VDNTFGAGGYFVQPLQYGADIVVHSATKWIGGHGTTVAGVIVDSGRFDWGKHA
FG01417.1 	KGVPVI0VDNTFGAGGYFIRPIDHGADIVVHSATKWIGGHGTTIGGVVVDSGKFDWGKNA
AN8277.1  	KGIPLV0VDNTFGAGGYFVRPIEHGADIVVHSATKWIGGHGTTIGGVVVDSGKFDWGKNA
          	**:*:: ***********::*:::*******************:.*::****:**** ::

MG07195.1 	KRFPQMVEPSEGYHGLKFWETFGPITFAIRVRVEVLRDLGACLSPFAAQQLILGLETLSL
NCU01652.1	DRFPQMVEPSEGYHGLKFWETFGPMAYITRVRVEILRDLGACLNPFAAQQLLLGLETLSL
FG01417.1 	SRFPQFHEPSEGYHGLKFYETFGNITFIIRARVEILRDLGSALNPFAAQQLLLGIETLSL
AN8277.1  	ARFPQFTQPSEGYHGLNFWETFGPIAFAIRVRVEILRDLGSALNPFAAQQLILGLETLSL
          	 ****: :********:*:**** :::  *.***:*****:.*.*******:**:*****

MG07195.1 	RAERHAQNALALARHLEKSPYVAWVSYPGLESHGSHENAKKYLKRGFGGVLSVGVKGGGA
NCU01652.1	RCERHTQNALALARYLEKSPYVSWVSYPGLESHGSHENAKKYLKRGFGGVLSFGVKGGAS
FG01417.1 	RAERHAQNALALAQYLEKSPYVSWVSYPGLESHPYHETAKKYLKRGFGGVLSFGVKG-GG
AN8277.1  	RAERHASNALALANWLKKNDHVSWVSYVGLEEHSSHEVAKKYLKRGFGGVLSFGVKGEAA
          	*.***:.******. *:*. :*:**** ***.*  ** **************.**** ..

MG07195.1 	AGSQVVDGFKMISNLAN~VGDSKTLAIHPWTTTHEQLSEQERISSGVTE~DLIRISVGTE
NCU01652.1	AGSQVVDGFKLISNLAN~VGDCKTLAIHPWSTTHEQLTDEEKISSGVTE~DLIRISVGIE
FG01417.1 	AGSEVVDGFKLISNLAN~VGDAKTLAIHPWSTTHEQLSDEEKRSSGVTE0DLIRISVGIE
AN8277.1  	VGSQVVDNFKLISNLAN2VGDSKTLAIHPWSTTHEQLTDQERIDSGVTE~DAIRISVGTE
          	.**:***.**:****** ***.********:******:::*: .***** * ****** *

MG07195.1 	HIDDIIADFEQSFKSAAATTKSTDGGD-AKGAPGKEDEAAPVVV
NCU01652.1	HIDDIIADFDQSFANAAATSAKEEGEKGLVVGQGKVEEAAPTVI
FG01417.1 	HIDDIIADFDQSFKAASDVTTKGEKKEIPLDDKEAEAPLAP---
AN8277.1  	HIDDIIADFEQSFAATFKVVRSA---------------------
          	*********:***  :  .  .
```
